# Lifetime Changes in Gut Microbiota and Metabolite Composition in High-Fat Diet-Induced Obesity in Apolipoprotein A-IV Gene Knockout Mice

**DOI:** 10.3390/biology14091278

**Published:** 2025-09-17

**Authors:** Natalia Zeber-Lubecka, Maria Kulecka, Aneta Balabas, Pawel Czarnowski, Kazimiera Pyśniak, Michalina Dąbrowska, Jerzy Ostrowski, Ewa E. Hennig

**Affiliations:** 1 Department of Gastroenterology, Hepatology and Clinical Oncology, Centre of Postgraduate Medical Education, 02-781 Warsaw, Poland; 2 Department of Genetics, Maria Sklodowska-Curie National Research Institute of Oncology, 02-781 Warsaw, Poland

**Keywords:** apolipoprotein A-IV, high-fat diet, microbiome, obesity, mouse model

## Abstract

Apolipoprotein A-IV (ApoA-IV) has been implicated in modulating the gut microbiota. However, chronic high-fat diet (HFD) consumption impairs ApoA-IV signaling and disrupts gut microbial balance, contributing to obesity and insulin resistance. This study aimed to investigate the role of ApoA-IV in shaping the gut microbiota and associated metabolic profiles throughout the lifespan of mice exposed to an HFD. Fecal samples were collected from ApoA-IV knockout (KO) and wild-type mice at five time points for microbiota and metabolite profiling using 16S rRNA gene sequencing and gas chromatography–mass spectrometry, respectively. Lifespan was longest in ApoA-IV-KO mice on a normal diet, while the HFD reduced survival across genotypes. Microbiota analysis revealed diet- and age-dependent shifts, including an elevated Firmicutes/Bacteroidota ratio, altered abundance of *Akkermansia* and reduced *Monoglobus* in ApoA-IV-KO mice on the HFD. Metabolic profiling showed a stronger impact of diet than genotype, with early and persistent increases in branched-chain amino acids and reductions in short-chain fatty acids (SCFAs). ApoA-IV deficiency modulated lifespan microbial and metabolic changes and shaped distinct responses to dietary stress. Despite age-related convergence in microbiota structure, genotype-specific differences in metabolite profiles and SCFA-producing bacteria correlations persisted into old age, demonstrating the lasting impact of ApoA-IV on host metabolic adaptation.

## Simple Summary

This study explored how the protein ApoA-IV affects the gut bacteria and metabolism in mice fed a high-fat diet over their lifespan. This study showed that diet had a stronger impact on gut microbes and metabolic changes than the absence of ApoA-IV. However, ApoA-IV deficiency still influenced how the body responded to long-term dietary stress. Even in old age, differences in metabolism and gut bacteria remained, showing that ApoA-IV plays a lasting role in how the body adapts to diet and aging.

## 1. Introduction

Glycoprotein apolipoprotein A-IV (ApoA-IV) is one of the most abundant plasma proteins, synthesized in response to dietary lipid absorption almost exclusively in the small intestine and in rodents additionally in the liver and hypothalamus [1]. A large number of studies indicate the participation of ApoA-IV in various physiological processes, such as lipid and glucose metabolism [2], satiety and food intake [3], the maintenance of energy homeostasis [4], anti-inflammation and anti-oxidation [5], platelet aggregation and thrombosis [6], disorders that may lead to various cardiometabolic diseases. Its protective role against atherosclerosis and diabetes has been repeatedly reported [7,8].

Human and rodent studies indicate that the production of ApoA-IV in the small intestine occurs immediately after high-fat diet (HFD) consumption [9]. Then, ApoA-IV is packaged onto TG-rich chylomicrons and secreted into intestinal lymph. Fat ingestion increases circulating ApoA-IV levels, which influences glucose homeostasis and the short-term regulation of satiety [10]. However, prolonged HFD consumption appears to disrupt this system, which becomes less sensitive to dietary lipid stimulation, and the initially significantly elevated ApoA-IV levels eventually decrease to control values [11]. Attenuated intestinal ApoA-IV response to chronic high-fat feeding may be related to increased circulating leptin levels, the secretion of which is stimulated by an HFD and certain hormones, including cholecystokinin (CCK) and insulin [12]. Central leptin, unlike its action in the intestine, enhances ApoA-IV expression in the hypothalamus via STAT3-dependent synergistic signaling [13].

ApoA-IV plays a key role in glycemic control and energy expenditure. ApoA-IV-deficient mice exhibited impaired glucose tolerance [14] and reduced diet-stimulated brown adipose tissue thermogenesis [4]. An injection of exogenous ApoA-IV administered to ApoA-IV knockout (KO) mice improved glucose tolerance by increasing glucose-dependent insulin secretion [14] and reducing hepatic gluconeogenesis [15]. Recent studies have demonstrated that HFD-induced ApoA-IV mediates beneficial effects on glucose and lipid metabolism in human liver and adipose tissues, such as reduced hepatic glucose production, enhanced lipoprotein clearance capacity, and elevated whole-body fatty acid oxidation [16].

ApoA-IV can directly stimulate CCK secretion, and both proteins work codependently to maintain control over food intake [17]. Since peripheral ApoA-IV cannot cross the blood–brain barrier, it requires the activation of vagal afferent neurons via the CCK1 receptor (CCK-1R)-dependent pathway to elicit satiety [10]. An HFD reduces the responsiveness of CCK-sensitive neurons and decreases the satiating capacity of both these proteins [18]. In obese humans, plasma ApoA-IV is elevated and its levels are higher in healthy individuals with obesity than in obese individuals with metabolic disorders [19]. Moreover, ApoA-IV concentration increases following bariatric surgery in diabetic obese patients [20]. Thus, impairments in intestinal and hypothalamic ApoA-IV production, for example, due to chronic HFD exposure, may attenuate the satiety feedback response, promoting sustained overeating and ultimately contributing to obesity through an energy imbalance, where caloric intake exceeds expenditure.

Growing evidence shows that various metabolic disorders, including diabetes and obesity, are associated with changes in the gut microbiome [21,22,23,24]. These alterations are generally detrimental and have been linked to the development and progression of type 2 diabetes, primarily through mechanisms involving impaired insulin sensitivity, chronic low-grade inflammation, and altered glucose metabolism [25,26]. Dysbiosis may also affect the production of microbial metabolites, including lipopolysaccharides, which activate inflammatory pathways and contribute to insulin resistance [27]. Environmental factors, including diet composition, antibiotic exposure, and aging, further modulate microbiome structure and function, influencing susceptibility to metabolic diseases [28].

Mouse intestinal microbiota are mainly composed of Bacteroidota (*Bacteroides*), Firmicutes (*Clostridium*, *Streptococcus*, *Lactobacillus*), Proteobacteria (*Enterobacteriaceae*) and Actinobacteria (*Streptomyces*, *Actinomyces*) [29]. Recent studies indicate changes in the composition of the gut microbiota in obese mice [30,31]. The colonization of germ-free mice with microflora from obese mice resulted in a significant increase in total body fat compared with colonization from lean animals [32]. Further studies showed that the transplantation of enterotoxigenic *Enterobacter* species from obese human donors induces obesity and insulin resistance in germ-free mice fed an HFD [33]. In turn, our own study demonstrated that the prolonged supplementation of HFD-fed mice with feces from mice fed a normal diet (ND) accelerated the onset of obesity [34].

The gut microbiota and its metabolites interact with the host and contribute to many metabolic functions, for example, by facilitating the digestion of nutrients. Dietary fiber and carbohydrates are fermented by intestinal microflora into short-chain fatty acids (SCFAs). SCFAs affect intestinal gluconeogenesis and lipid formation, regulate host energy metabolism, improve bowel function and protect against pathogenic microorganisms [35,36]. Some SCFAs, such as acetic acid, butyric acid, and propionic acid (90–95% of SCFAs), are produced exclusively by the gut microbiota [37]. On the other hand, the gut microbiota itself synthesizes a relatively small proportion of amino acids [38] but utilizes amino acids derived from dietary proteins and provides them to the host [39]. The amino acid composition in the gastrointestinal tract depends primarily on the content of branched-chain amino acids (BCAAs): valine, leucine and isoleucine [40]. Both SCFAs and amino acids are involved in obesity development [41,42,43]. Higher SCFA concentrations were shown in the feces of obese humans, which may be due to increased SCFA production [44]. In turn, BCAA levels correlated with insulin resistance, which is associated with high-fat consumption [42].

Aging is associated with changes in the microbiome regarding the structure and functions of the bacterial community, with older individuals being characterized by reduced microbial diversity and altered proportions between different taxa [45]. These changes may negatively impact host immunity and metabolism [46], potentially shortening lifespan due to impaired physiological homeostasis. In the present study, we investigated, for the first time, changes in gut microbiota composition and the associated metabolite profile across the lifespan of mice. ApoA-IV-deficient mice were employed to elucidate the role of ApoA-IV in microbiome and metabolic alterations contributing to the development of HFD-induced obesity.

## 2. Materials and Methods

### 2.1. Development of ApoA-IV Knockout Mice and Maintenance of Animals

A model of ApoA-IV-KO mice was developed by Professor Jan L. Breslow (Rockefeller University, New York, NY, USA) [47] and kindly provided by Professor Patrick Tso (University of Cincinnati, Cincinnati, OH, USA). Briefly, a targeting vector was designed to replace exon 1 and intron 1 of the endogenous *ApoA4* gene with a neomycin resistance gene. This construct was electroporated into 129S4/SvJae-derived J1 embryonic stem cells, which were further injected into C57BL/6J blastocysts. Chimeric mice were backcrossed onto a C57BL/6 genetic background for many generations. In homozygous mice, no detectable *ApoA4* transcript was confirmed by RNA sequencing and no stable protein in plasma was demonstrated by Western blot analysis using ApoA4 (1D6B6) mouse antibody (Cell Signaling Technology Inc., Danvers, MA, USA).

Male ApoA-IV-KO and C57BL/6 wild-type (WT) mice were bred under standard laboratory conditions at the core animal facility of the Maria Sklodowska-Curie National Research Institute of Oncology in Warsaw. Animals were maintained in climate-controlled rooms with regulated humidity (55 ± 10%) and temperature (21 ± 2 °C), a 12 h light/dark cycle, and ad libitum access to food and water. Health monitoring was performed in accordance with the recommendations of the Federation of European Laboratory Animal Science Associations (FELASA), including screening for parasites, bacteria, and viruses.

### 2.2. Experiment Design

**Figure 1:**
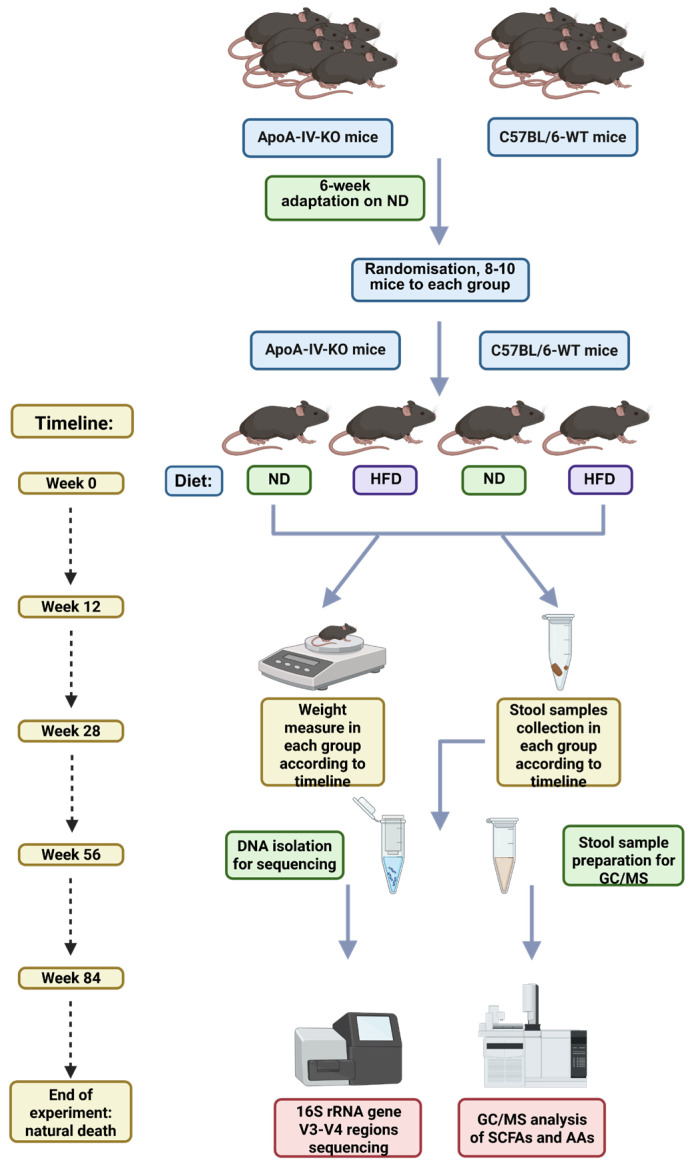
Graphical presentation of the experiment design. GC/MS; gas chromatography–mass spectrometry, AA; amino acid, HFD; high-fat diet, KO; knockout, ND; normal diet, SCFA; short-chain fatty acid, WT; wild type.

During the first six weeks of life, both ApoA-IV-KO and WT mice were fed an ND (10% of calories from fat) containing 10% fat, 22% protein, and 51% carbohydrate (Labofeed H, Feed Factory Morawski, Kcynia, Poland). At six weeks of age, mice of both genotypes were randomly divided each into two groups (8–10 mice per group) and then either continued to be fed an ND or started on an HFD (30% of calories from fat) containing 30% fat, 22% protein, and 51% carbohydrate (Labofeed H, Feed Factory Morawski, Kcynia, Poland). Animals were housed in the same room and under the same conditions throughout their lives and raised until natural death. At week 0 (just before the start of HFD feeding) and at weeks 12, 28, 56 and 84 after the start of experiment, animals from all groups were individually weighed and samples of feces were collected from each mouse and stored at −80 °C until use (Figure 1).

### 2.3. DNA Extraction and 16S rRNA Gene Sequencing

Genomic DNA was extracted from fecal dropping samples using a QIAamp Fast DNA Stool Kit protocol (Qiagen GmbH, Hilden, Germany). The quality and quantity of the purified DNA were measured using a NanoDrop 2000/2000c spectrophotometer (Thermo Fisher Scientific, Waltham, MA, USA) and a fluorometric-based method using a Qubit dsDNA HS Assay Kit (Thermo Fisher Scientific, Waltham, MA, USA), respectively. A metagenomic analysis of fecal microflora was performed using Illumina (San Diego, CA, USA) technology and protocols by the taxonomic identification of bacteria based on prepared sequencing libraries targeting the variable V3 and V4 regions of the bacterial 16S rRNA gene. Paired-end sequencing was performed on an Illumina MiSeq system with a 2 × 300 bp run.

### 2.4. Gas Chromatography–Mass Spectrometry Metabolite Analysis

Selected metabolites extracted from fecal dropping samples and derivatized as described previously [48] were subjected to gas chromatography–mass spectrometry (GC/MS) analysis on an Agilent 7000D Triple Quadrupole mass spectrometer coupled with a 7890 GC system and G4513A autosampler (Agilent Technologies, Santa Clara, CA, USA), with a VF-5ms column (30 m, 0.25 mm, and 0.50 µm). Calibration standards of SCFAs (formic acid, acetic acid, propionoic acid, isobutyric acid, butanoic acid, pentanoic acid, isocaproic acid and hexanoic acid) and amino acids (alanine, glycine, valine, leucine, isoleucine, proline, methionine, phenylalanine and glutamic acid) were obtained from Sigma-Aldrich (St. Louis, MO, USA). MS data were collected in full-scan mode from m/z 15 to 300 at a rate of 4 scans/s. MassHunter software (Agilent Technologies, Santa Clara, CA, USA) v10.1 was used for analysis.

### 2.5. Data Analysis

The statistical differences in mean life expectancy were assessed using multiple unpaired *t*-tests, while body weight was analyzed by three-way ANOVA. To control for false discovery rate, the two-stage step-up procedure described by Benjamini et al. [49] was applied. Sequence preprocessing was conducted using Mothur [50] v1.43. Contigs were created from paired-end reads and sequences with ambiguous bases and a homopolymer length higher than 8 were removed. Chimeric sequences were removed using the vsearch [51] algorithm and Silva Gold reference file (https://mothur.org/wiki/silva_reference_files/, accessed on 13 January 2025). Taxonomic classification was then assigned to the remaining 16S rRNA sequences using Wang’s method and Silva [52] database v138 as a reference. The α-diversity metrics were also computed with Mothur software. Differences in α-diversity indices and Firmicutes to Bacteroidota (F/B) ratios were assessed with the Mann–Whitney *U*-test. β-diversity was measured with Aitchison distances, after replacing zeros using zCompositions [53] package v1.4 based on Bayesian-multiplicative replacement, and visualized by principal coordinate analysis (PCoA) plots. The statistical significance of the groupings related to β-diversity was assessed with PERMANOVA. Bacterial taxon differential abundance between groups was estimated with LinDA [54] using default parameters, with *p*-values adjusted (*p*_adj_) by the Benjamini–Hochberg procedure [55]. Differences in metabolite concentration between groups were estimated with the Mann–Whitney *U*-test.

Regularized canonical correlation analysis (rCCA) was performed on identified bacterial taxa and metabolites using the ridge method with parameter tuning as described in the mixOmics [56] v6.22 tutorial (http://mixomics.org/case-studies/rcca-nutrimouse-case-study/, accessed on 4 March 2025). The correlation structure was visualized with the complexHeatmap [57] package v2.14. Bacterial species were clustered using the “ward. D2” method in the base R hclust function. The optimal number of modules was selected using the dynamicTreeCut [58] package v1.63, with a minimum cluster size of 8. On the visualization, only bacteria with more than 5 correlation coefficients with values higher than 0.1 were presented.

## 3. Results

In the following study, we investigated the lifetime changes in the gut microbiota and metabolite composition in ApoA-IV-KO mice compared to C57BL/6 WT mice, fed the ND or HFD. Fecal samples were collected at five experimental time points (Figure 1) throughout the lifespan of mice and used for 16S rRNA gene metagenomic sequencing and GC/MS analysis.

### 3.1. Mice Characteristics

**Figure 2:**
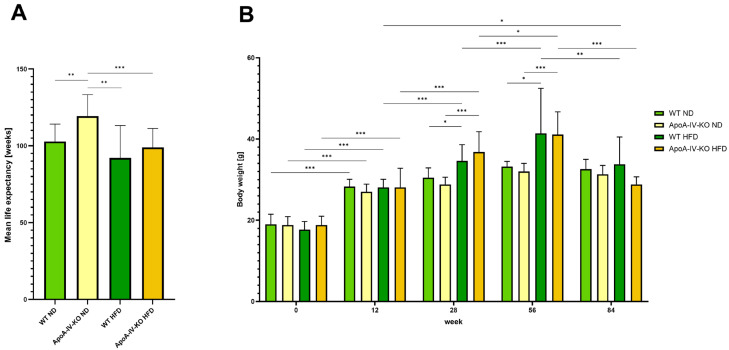
The mean life expectancy of mice (**A**), and over the lifespan changes in the mouse body weight (**B**). Statistical significance values: * *q* < 0.05, ** *q* < 0.001, *** *q* < 0.0001. HFD; high-fat diet, KO; knockout, ND; normal diet, WT; wild type.

ApoA-IV-KO and C57BL/6 WT mice were bred on either an HFD or ND until natural death. As shown in Figure 2A, ND-fed ApoA-IV-KO mice lived the longest on average, compared to the other groups. The mean lifespan of these mice (119.3 weeks ± 14.0) was significantly (*q* < 0.001) longer than that of WT mice fed an ND (102.8 ± 11.3), as well as ApoA-IV-KO and WT mice fed an HFD (98.9 ± 12.4 and 92.1 ± 21.1, respectively). Interestingly, ND-fed ApoA-IV-KO mice lived significantly (*q* = 7.7 × 10^−5^) longer than those fed the HFD, whereas the lifespan of WT mice did not differ significantly by diet. A similar trend to ApoA-IV-KO mice could be observed.

Each mouse was weighed at all time points of the experiment. As expected, HFD-fed mice, regardless of genotype, gained significantly more weight than ND-fed mice (Figure 2B), although this difference was clearly noticeable from week 28; at week 12, no differences were observed in the comparisons between the HFD and ND for both genotypes. After the initial weight gain (week 12) in mice fed both diets, no further increase in body mass was observed in the ND group, whereas in the HFD group, body weight increased significantly between weeks 12 and 28 and between weeks 28 and 56, regardless of genotype. At week 84, significant weight loss was observed in HFD-fed mice (week 84 vs. week 56) of both genotypes, such that the weight of mice from all groups did not differ significantly, regardless of diet. Furthermore, the weights of the ApoA-IV-KO mice on the HFD at week 84 decreased to the level observed at week 12.

### 3.2. Metagenomic Sequencing

An average of 60,782 contigs were generated per sample (median 61,040). Among all samples, five phyla accounted for more than 99% of the microbiota present, i.e., Bacteroidota, Firmicutes, Verrucomicrobiota, Proteobacteria and Actinobacteriota, in order of abundance. Out of 196 identified families, 32 were present in more than 0.01% reads. The five most abundantly represented families were *Muribaculaceae*, *Lachnospiraceae*, *Oscillospiraceae*, *Rikenellaceae* and *Akkermansiaceae*.

#### 3.2.1. Microbiota Diversity and Ratio of Firmicutes to Bacteroidota

**Figure 3:**
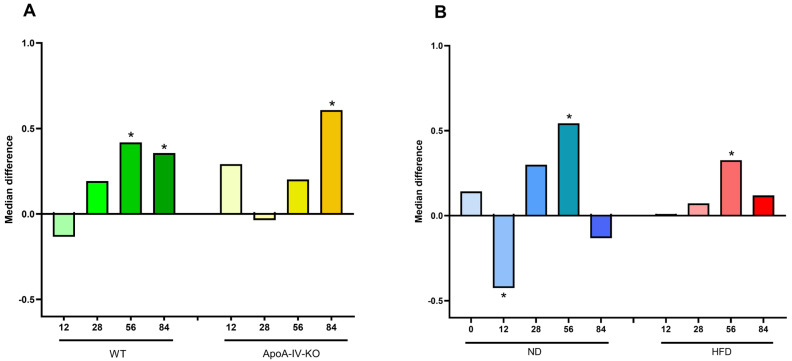
Microbiota α-diversity assessed by the Shannon index. (**A**) Median value differences in α-diversity between HFD and ND groups, calculated separately for WT and ApoA-IV-KO mice at each time point. (**B**) Median value differences between ApoA-IV-KO and WT mice, calculated separately for ND and HFD groups at each time point. Mann–Whitney *U*-test assessed statistical significance (* *p* < 0.05). HFD: high-fat diet; KO: knockout; ND: normal diet; WT: wild type.

**Figure 4:**
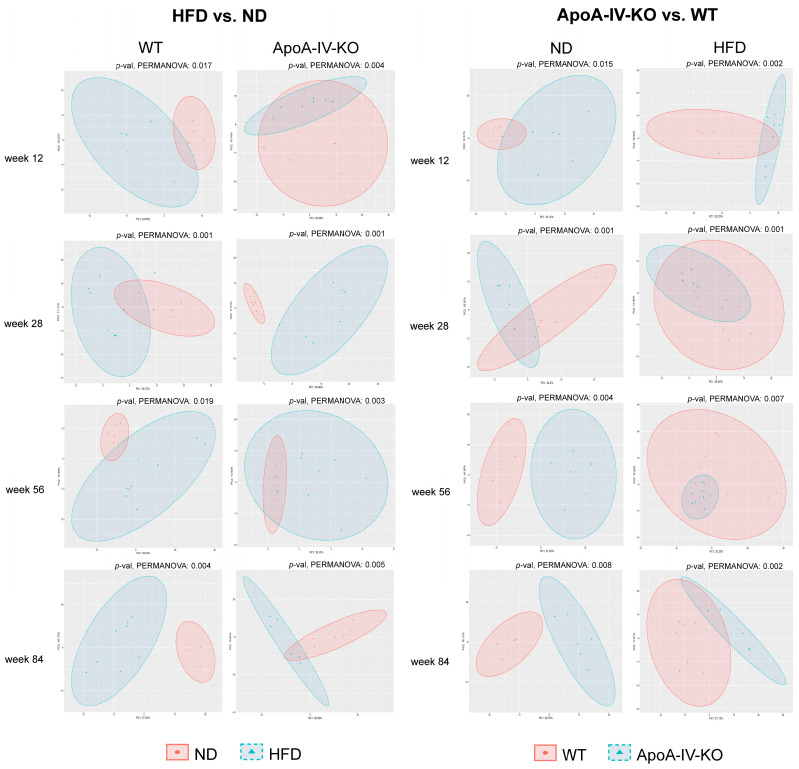
Microbiota β-diversity visualized using principal coordinate analysis (PCoA) based on Aitchison distances. Group differences were assessed using the PERMANOVA test. HFD: high-fat diet; KO: knockout; ND: normal diet; WT: wild type.

The structure of the gut microbiota community among fecal samples was evaluated using the Shannon and Chao indices for α-diversity (a marker of bacterial evenness and/or richness) and PCoA of Aitchison distances for β-diversity (a marker of diversity between samples). The analyses were performed at the family level. Bacterial diversity measured by the Shannon index tended to increase in older WT and ApoA-IV-KO mice fed an HFD compared to the corresponding mice fed an ND (Figure 3A). The Shannon index was also higher in ApoA-IV-KO mice than in WT mice at week 56, fed both the ND and HFD, but lower at week 12 when fed the ND (Figure 3B). However, the Chao index at week 12 was higher for HFD-fed ApoA-IV-KO mice than for those fed the ND (*p* = 6.2 × 10^−3^), which was the only significant difference in this index from all comparisons (see Supplementary Figures S1 and S2 for all Shannon and Chao comparisons). In turn, significant differences in β-diversity were found between all analyzed groups compared both in terms of genotype and diet (Figure 4).

**Figure 5:**
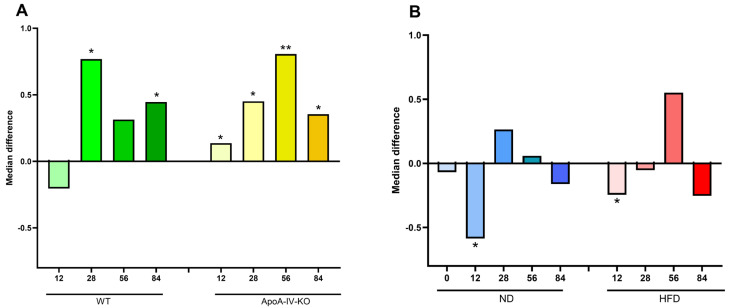
Differences in the Firmicutes to Bacteroidota ratio assessed as median differences across time points. (**A**) Median value differences between HFD and ND groups, regardless of genotype. (**B**) Median value differences between ApoA-IV-KO and WT mice, regardless of diet. Statistical significance: * *p* < 0.05, ** *p* < 0.001. HFD: high-fat diet; KO: knockout; ND: normal diet; WT: wild type.

The F/B ratio was calculated to compare the changes in the abundance of these two bacterial phyla in the gut microbiomes throughout the lifespan of the mice. As shown in Figure 5A, the F/B ratio was significantly higher in HFD-fed ApoA-IV-KO mice at all time points and in WT mice at weeks 28 and 84 compared to age- and strain-matched mice fed an ND. However, at the 12-week time point, the F/B ratio was significantly lower in ApoA-IV-KO mice than in WT mice on both the HFD and ND (Figure 5B). The results of all individual comparisons are shown in Supplementary Figure S3.

#### 3.2.2. Pairwise Comparative Analyses of Gut Microbiome Composition

**Figure 6:**
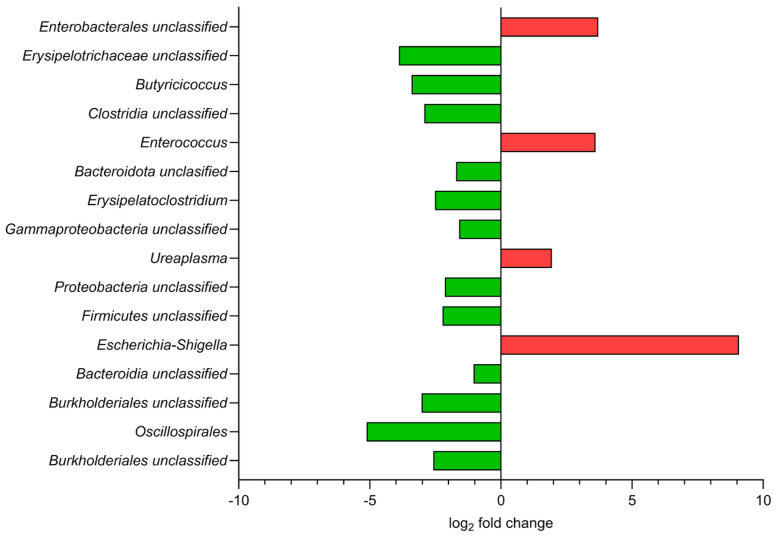
Microbiome composition differences at the baseline of the experiment (week 0). Relative abundance of bacterial genera in ApoA-IV-KO mice compared to WT mice, expressed as log_2_ fold change.

To assess variation in the gut microbiome over the life course of the mice, the relative abundance of bacteria at the genus taxon level was compared between mouse strains and diets at all experimental time points. We identified 16 bacterial genera that showed a significant difference (*p*_adj_ < 0.05) in abundance between ApoA-IV-KO and WT mice at the starting point of the experiment (week 0, when all mice were still fed an ND), of which four and 12 genera were over- and under-represented, respectively (Figure 6). The differentially represented bacteria belonged mainly to free classes: Gammaproteobacteria (Proteobacteria), Bacilli and Clostridia (both belonging to Firmicutes). The most significant difference was the lower abundance of unclassified Burkholderiales (*p*_adj_ = 2.1 × 10^−4^) and Oscillospirales (*p*_adj_ = 4.6 × 10^−4^) and the higher abundance of *Escherichia-Shigella* (*p*_adj_ = 1.3 × 10^−3^) and *Ureaplasma* (*p*_adj_ = 2.6 × 10^−2^) in ApoA-IV-deficient mice than in WT mice, suggesting a potential shift towards dysbiosis.

**Figure 7:**
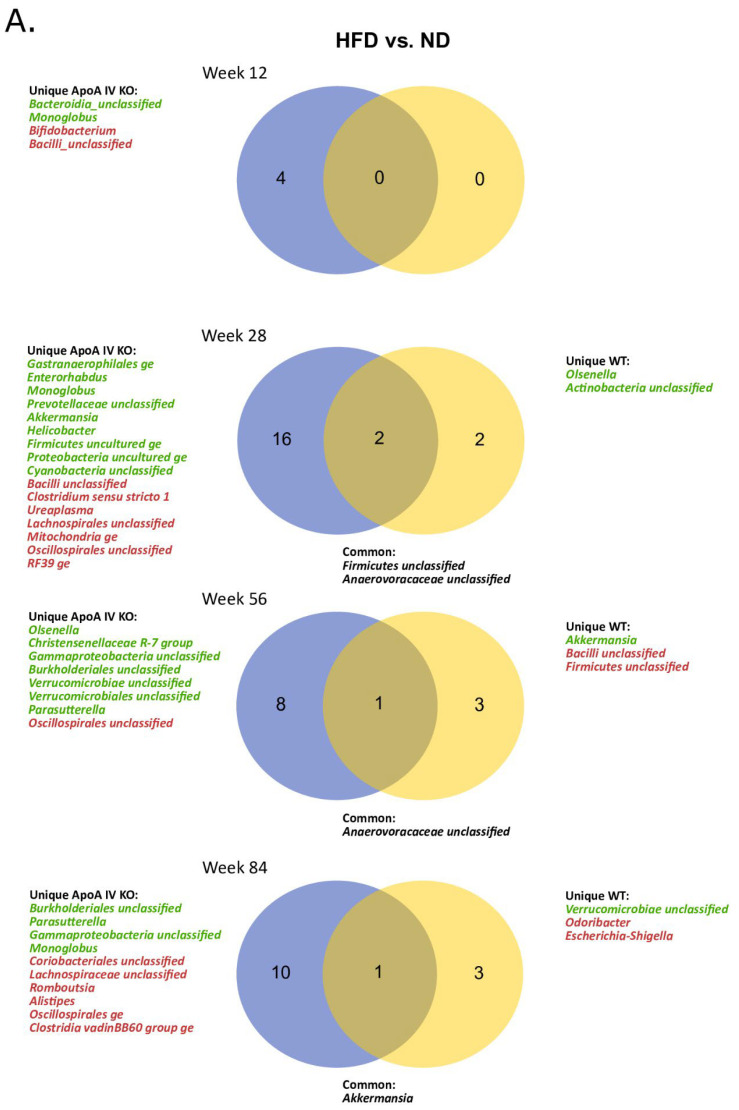

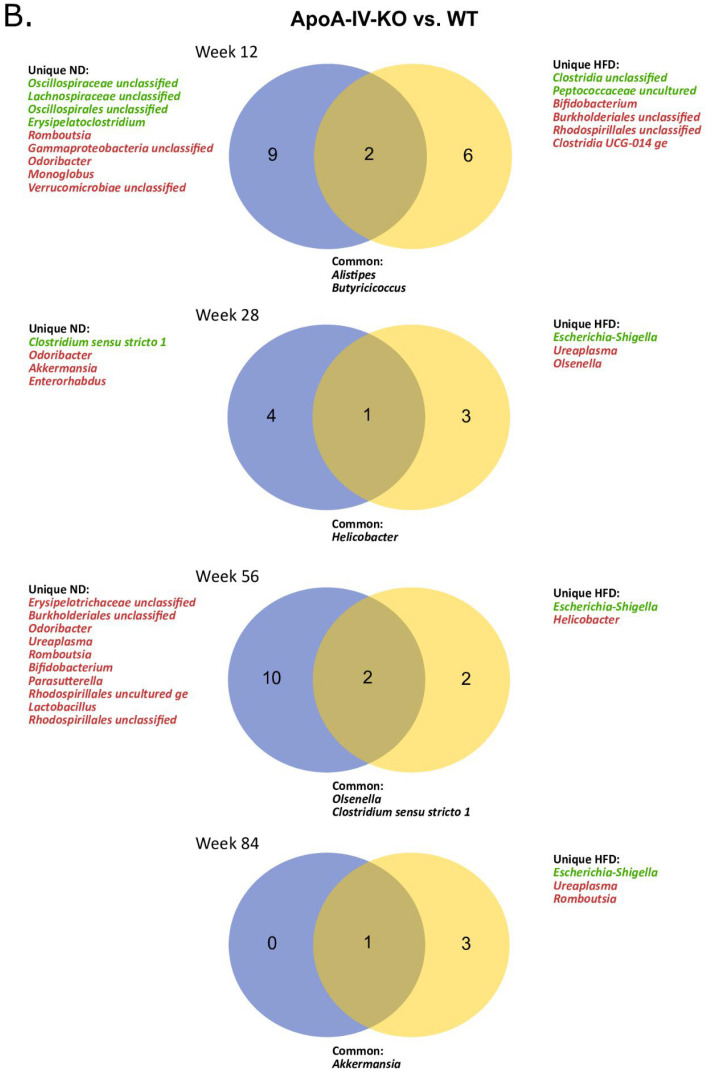
Venn diagrams illustrating shared and genotype- or diet-specific changes in microbiota composition at the genus level across experimental time points. Green indicates decreased and red indicates increased abundance of the respective genera in HFD-fed vs. ND-fed mice (**A**) and in ApoA-IV-KO vs. WT mice (**B**). Statistical significance *p*_adj_ < 0.05. HFD: high-fat diet; KO: knockout; ND: normal diet; WT: wild type.

The pairwise comparisons and further analysis of Venn diagrams indicated differentially abundant bacterial genera that were either common or unique to the mouse genotype or diet. Detailed data from all comparisons are provided in Supplementary Tables S1–S4. As shown in Figure 7A, HFD feeding was associated with significantly greater alterations in the gut microbiome of ApoA-IV-deficient mice compared to WT mice. At weeks 12, 28, 56, and 84, 4, 16, 8, and 10 genera, respectively, showed uniquely altered abundance in ApoA-IV-KO mice, while only 0, 2, 3, and 3 genera were affected in WT mice. The most consistently observed was the lower representation of *Monoglobus* in the HFD-fed ApoA-IV-deficient mice compared to the ND-fed mice (weeks 12, 28 and 84). The greatest difference was observed at week 28 in ApoA-IV-KO mice, showing 16 genera with uniquely changed abundance, of which nine and seven were down- and over-represented, respectively.

The HFD did not substantially affect gut microbiome composition during the initial weeks of feeding compared to the ND. At week 12, no bacterial genera differentiated WT mice, while four genera distinguished ApoA-IV-deficient mice fed an HFD from those fed an ND, based on log_2_ fold change in relative abundance. Interestingly, only a few bacterial genera were common to both strains in this comparison, as down-represented unclassified *Anaerovoracaceae* (week 28 and 56) and *Akkermansia* (week 84), however the relative abundance of the latter one was increased in ApoA-IV-KO mice and decreased in WT mice. Furthermore, *Akkermansia* was uniquely decreased in ApoA-IV-KO mice at week 28 and in WT mice at week 56. Similarly, an increased abundance of unclassified *Bacilli* and decreased abundance of unclassified *Verrucomicrobiae* were observed in both strains, but with a 28-week time shift, with the change occurring earlier in the ApoA-IV-KO strain (weeks 28 and 56, respectively).

Overall, more unique differences were observed between the microbiomes of ApoA-IV-KO and WT mice when fed an ND as opposed to an HFD. At weeks 12, 28, and 56, 9, 4, and 10 genera, respectively, showed differential abundance, as shown in Figure 7B. However, no differences were shown on the ND at week 84. Interestingly, *Odoribacter* was the genus whose relative abundance increase was unique for ND-fed ApoA-IV-KO mice, consistently at weeks 12, 28 and 56. The highest number of unique microbiome differences in HFD-fed mice was observed in young animals at week 12, with six differentiating genera. At subsequent time points, primarily a decreased representation of genus *Escherichia-Shigella* (weeks 28, 56, and 84) and increased representation of *Ureaplasma* (weeks 28 and 84) were observed. Surprisingly, the relative abundance of *Akkermansia* in ApoA-IV-KO mice was higher than in WT mice on the ND at week 28 but lower at week 84. In contrast, *Akkermansia* was over-represented in ApoA-IV-deficient mice on the HFD at week 84.

### 3.3. Metabolite Composition

**Figure 8:**
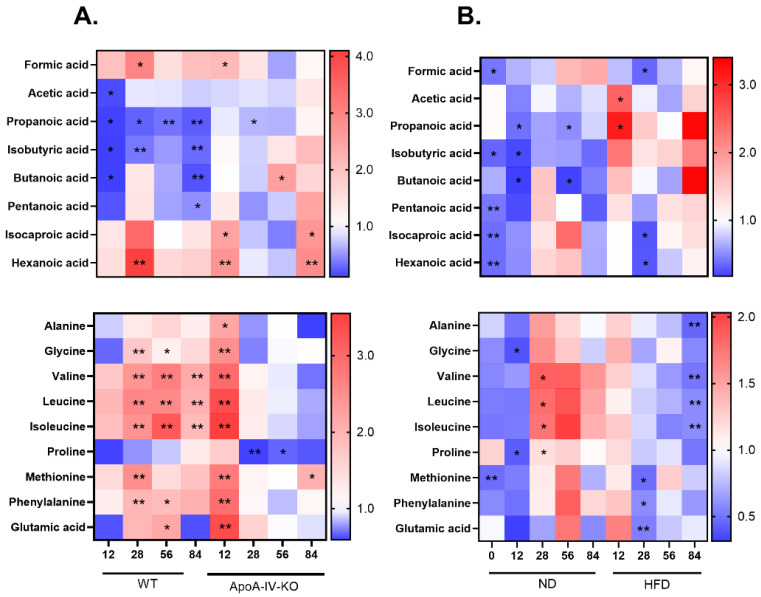
The heatmap showing fold change in difference in median concentration of SCAFs and amino acids in HFD vs. ND group (**A**) and in ApoA-IV-KO vs. WT mice (**B**) at all experimental time points. Statistical significance: ** *p_adj_* < 0.05, * *p* < 0.05. HFD: high-fat diet; KO: knockout; ND: normal diet; WT: wild type.

GC/MS-based analyses of metabolite concentrations in fecal samples revealed that all but two (formic acid and acetic acid) measured SCAFs and amino acids distinguished between mouse genotypes or diets at at least one experimental time point, with a *p*_adj_ < 0.05 significance value. Heatmaps showing the fold change in the difference in SCAF and amino acid median concentrations are presented in Figure 8. Noticeably, more changes in metabolite concentrations were observed in the comparison between diets than between genotypes.

At time point 0, the levels of three SCAFs (pentanoic, isocaproic, and hexanoic acids) and methionine were lower in ApoA-IV-deficient mice compared to WT mice (Figure 8B), which is consistent with the observed differences in their gut microbiomes (Figure 6). As illustrated in Figure 8A, the greatest changes in metabolite composition, mainly increases in amino acid levels, in HFD-fed compared to ND-fed ApoA-IV-KO mice were observed at week 12, whereas only minor differences were detected at the remaining time points. In contrast, in HFD-fed vs. ND-fed WT mice at week 12, none of the metabolites differentiated with significance *p*_adj_ < 0.05 and only four SCAFs (acetic, propanoic, isobutyric and butanoic acids) showed reduced concentrations at *p* < 0.05. Most differences emerged at week 28 and persisted in the subsequent weeks, including a decrease in propanoic, isobutyric, and butanoic acid levels, as well as an increase in three BCAAs, glycine, methionine, and phenylalanine levels.

Major differences between ApoA-IV-KO and WT mice fed an HFD were observed at week 84, including decreased concentrations of three BCAAs and alanine (Figure 8B). Additionally, the level of glutamic acid was decreased at week 28. There were no significant differences at week 56. At week 12, acetic and propanoic acid concentrations were higher in HFD-fed ApoA-IV-KO mice than in WT mice, whereas on the ND, the levels of propanoic, isobutyric and butanoic acids and glycine and proline were lower, but only at *p* < 0.05 significance. Similarly, lower levels of propanoic and butanoic acids were observed at week 56 and lower levels of isobutyric acid at week 84. In turn, at week 28, the concentration of four amino acids, including three BCAAs, was higher in ApoA-IV-deficient mice than in WT mice (*p* < 0.05), whereas no differences in amino acid levels were observed between aged mice of both strains fed the ND (weeks 56 and 84).

### 3.4. The Correlation Analysis on Identified Bacterial Taxa and Metabolite Concentrations

**Figure 9:**
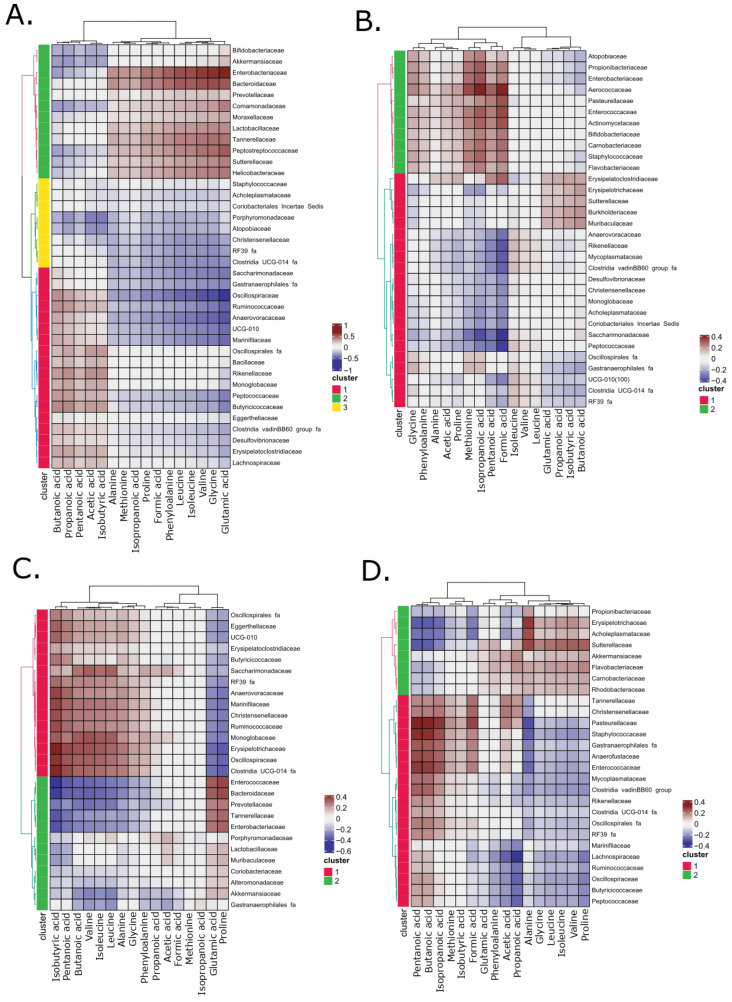
Heatmaps derived from pairwise correlations between bacterial abundances and measured metabolite concentrations, identified based on the first and second canonical variates from regularized canonical correlation analysis (rCCA). Panels show data from ND-fed WT mice (**A**), HFD-fed WT mice (**B**), ND-fed ApoA-IV-KO mice (**C**), and HFD-fed ApoA-IV-KO mice (**D**). Statistically significant correlation coefficients (*p*_adj_ < 0.05) are presented and color-coded.

Pairwise correlations between the abundance of identified bacterial family taxa and metabolite concentrations in fecal samples from all four study groups were evaluated based on the first two canonical variates derived from rCCA. The correlation structure and magnitude of individual values within the obtained datasets were visualized in heatmaps (Figure 9). The analysis revealed that 38, 32, 27 and 27 of bacterial taxa had a significant correlation with metabolites for ND- and HFD-fed WT mice and ND- and HFD-fed ApoA-IV-KO mice, respectively.

In ND-fed WT mice, the correlation patterns formed three distinct bacterial clusters and two metabolite clusters. The first metabolite cluster included five SCAFs (butanoic, propanoic, pentanoic, acetic, and isobutyric acids), while the second comprised the remaining SCAFs and all amino acids. Bacterial cluster 1 contained bacteria positively correlated with SCAFs from the first metabolic cluster and negatively with metabolites from the second one. Especially *Oscillospiraceae* positively correlated with butanoic and propanoic acids and negatively with BCAAs and glutamic acid. The opposite correlations were observed in bacterial cluster 2, where *Enterobacteriaceae* and *Bacteroidaceae* expressed the strongest positive correlation with BCAAs and glutamic acid, whereas *Akkermansiaceae* and *Bifidobacteriaceae* negatively correlated with SCAFs from the first cluster. For the HFD-fed WT mice, two bacterial clusters and two metabolite clusters were formed. Overall, very weak correlations were observed for metabolites from the second cluster, including BCAAs, glutamic acid, and three SCAFs (propanoic, isobutyric, and butanoic acids). The strongest correlations were shown for formic, isocaproic and pentanoic acids: positive relationships with *Aerococcaceae* and negative with *Saccharimonadaceae.*

Similarly, in ND-fed ApoA-IV-KO mice, two distinct clusters were formed for both bacterial taxa and metabolites based on their correlation patterns. In the first cluster of metabolites, the strongest correlations were observed for isobutyric, and pentanoic acids, positively correlated with bacteria from cluster 1, like *Erysipelotrichaceae* and *Oscillospiraceae,* but negatively correlated with bacteria from cluster 2, including *Enterococcaceae*, *Enterobacteriaceae* and *Bacteroidaceae*. Also, strong positive relationships were observed for BCAAs and butanoic acid with *Saccharimonadaceae*, *Erysipelotrichaceae* and *Monoglobaceae* from cluster 1 and a negative correlation with *Bacteroidaceae*, *Prevotellaceae* and *Akkermansiaceae* from cluster 2. The strength of associations between bacteria and metabolites from the second cluster was generally weak or absent, except for proline and glutamic acid, which showed opposite correlation patterns compared to metabolites from the first cluster.

Also, in HFD-fed ApoA-IV-KO mice, two clusters each were formed for bacteria and metabolites. The strongest relationships regard pentanoic, butanoic, isocaproic and formic acids from the first cluster, which were strongly negatively correlated with *Erysipelotrichaceae*, *Sutterellaceae* and *Acholeplasmataceae* and positively correlated with *Pasteurellaceae*, *Staphylococcaceae* and *Enterococcaceae.* From the second metabolic cluster, propanoic and acetic acids showed negative relationships with *Lachnospiraceae.* In turn, alanine negatively corelated with *Pasteurellaceae* and *Staphylococcaceae* and positively correlated with *Erysipelotrichaceae*, *Acholeplasmataceae* and *Sutterellaceae.* Additionally, *Sutterellaceae* expressed a strong positive relationship with BCAAs and proline.

## 4. Discussion

Gut dysbiosis and metabolic disturbances have been recognized as key pathophysiological mechanisms in the development of obesity and metabolic diseases [59]. In recent years, increasing attention has been paid to the interaction between gut microbiota, diet, and genetic factors, including proteins involved in lipid transport and appetite regulation [60]. ApoA-IV is one such protein, whose role in metabolic homeostasis has been partially described; however, its impact on the gut microbiota remains poorly understood [10].

The ApoA-IV-KO mouse model was originally developed to investigate the physiological functions of ApoA-IV [16]. Beyond its canonical roles in lipid transport and appetite regulation, ApoA-IV has emerged as a multifunctional protein implicated in anti-inflammatory signaling, oxidative stress modulation, and insulin sensitivity [7]. The ApoA-IV-KO model provides a valuable platform for investigating the complex interplay between host metabolism, immune responses, and environmental factors such as diet [10]. Despite its relevance, the impact of ApoA-IV deficiency on gut microbiota composition and function has not been previously addressed in a longitudinal context.

This study is the first to investigate changes in gut microbiota composition and metabolite profiles across key stages of the murine lifespan, considering the combined effects of an HFD and ApoA-IV deficiency. It has been shown that aging leads to reduced microbial diversity, a decline in SCFA-producing bacteria, and an increase in opportunistic pathogens, which collectively promote chronic inflammation and metabolic dysfunction [61]. These age-related microbial shifts have been consistently observed in both murine models and human cohorts, where aging is associated with a loss of beneficial taxa such as *Lachnospiraceae* and *Ruminococcaceae* and an enrichment of pro-inflammatory Proteobacteria [62]. Previous longitudinal studies have shown that the aging gut microbiota exhibits reduced metabolic flexibility, characterized by diminished SCFA production and altered amino acid metabolism, particularly involving BCAAs [2].

We observed the longest lifespan in ApoA-IV-KO mice fed the ND, which may suggest some beneficial effect of ApoA-IV deficiency, especially under physiological conditions. Under HFD conditions, mouse lifespan was reduced regardless of genotype, confirming the detrimental impact of chronic fat overload on systemic homeostasis. Gut microbiota analysis revealed that five dominant bacterial families, *Muribaculaceae*, *Lachnospiraceae*, *Oscillospiraceae*, *Rikenellaceae*, and *Akkermansiaceae*, were most abundantly represented in all groups of samples. *Muribaculaceae* and *Rikenellaceae* are associated with polysaccharide metabolism and SCFA production, while *Lachnospiraceae* and *Oscillospiraceae* are known butyrate producers that support gut barrier integrity and exert anti-inflammatory effects [63,64]. *Akkermansia muciniphila*, representing *Akkermansiaceae*, degrades mucin and supports glucose metabolism and gut barrier function [65]. In our study, the abundance of *Akkermansia* in ApoA-IV-KO mice changed dynamically during long-term HFD consumption, decreasing in middle-aged mice and increasing again in old age. No such effect was observed in aged WT mice, suggesting that microbiome remodeling in response to chronic metabolic stress may be specific to ApoA-IV deficiency. This dynamic behavior of *A. muciniphila* may reflect its dual role as both a marker and modulator of host metabolic health [66]. Previous studies have shown that *A. muciniphila* abundance declines with age and metabolic dysfunction, but its restoration, either through dietary interventions or direct supplementation, can improve gut barrier integrity, reduce systemic inflammation, and enhance insulin sensitivity [67,68,69]. In short-term HFD feeding, a negative correlation was found between *Akkermansia* and body weight gain in C57BL/6J mice [70]. The most consistently observed in HFD-fed ApoA-IV-KO mice was lower representation of *Monoglobus* than in ND-fed mice. *Monoglobus* belonging to *Ruminococcaceae* is a beneficial bacterium that degrades intestinal pectin, supports the production of SCAFs and regulates intestinal immune function [71,72]. Bárcena et al. demonstrated that microbiota transplantation from young animals to progeroid mice extended lifespan and improved metabolic parameters, associated with increased *A. muciniphila* and restored bile acid metabolism [73]. Similar shifts, reduced Proteobacteria and increased Verrucomicrobia, have been reported in long-lived humans, linked to improved gut barrier function and reduced inflammation [32,73].

The F/B ratio was elevated in ApoA-IV-KO mice on an HFD at all time points compared to those on an ND, consistent with previous reports linking increased F/B ratios to obesity and enhanced microbial energy harvesting efficiency [74]. The observed shifts toward increased Firmicutes and decreased Bacteroidota under an HFD are consistent with classical observations by Turnbaugh et al. in obese mice [32]. The increase in the abundance of the phylum Firmicutes was mainly associated with the proliferation of the *Erysipelotrichaceae* family [75]. Interestingly, the F/B ratio was lower in ApoA-IV-deficient mice than in WT mice at week 12, especially on the ND. This was complemented by lower bacterial biodiversity as shown by the Shannon index, suggesting that adverse changes in the microbiomes of ApoA-IV-KO mice occur later than in WT mice. While our study highlights delayed and dynamic microbiome remodeling in ApoA-IV-deficient mice under dietary stress, previous studies have described a range of metabolic outcomes linked to ApoA-IV deficiency. For instance, Qu et al. [76] observed that female ApoA-IV-KO mice developed obesity and insulin resistance under an HFD, despite unchanged food intake, which they attributed to reduced energy expenditure. Pence et al. [4] demonstrated impaired thermogenesis in the brown adipose tissue of ApoA-IV-KO mice, suggesting a role for ApoA-IV in maintaining energy balance. Additionally, Wang et al. [2] showed an age-independent modulation of hepatic metabolic gene expression in ApoA-IV-deficient rats. Although these studies do not directly address microbiome dynamics, they provide valuable context for understanding the broader physiological impact of ApoA-IV deficiency. Our findings offer a novel perspective by focusing on lifespan-associated changes in gut microbiota composition in ApoA-IV-deficient mice, an area that, to our knowledge, has not been previously explored. This microbiome-centered approach complements existing metabolic studies and contributes to a more comprehensive understanding of how ApoA-IV deficiency interacts with aging and dietary stress.

Remarkable transient microbial changes were observed, including a reversal of the initial state at week 0, when *Escherichia-Shigella* dominated in ApoA-IV-KO mice on the ND, while unclassified *Burkholderiales* and unclassified *Erysipelotrichaceae* were reduced. At later time points, we observed a consistent decline in *Escherichia-Shigella* when feeding the HFD and an increase in unclassified *Burkholderiales* and unclassified *Erysipelotrichaceae* with the ND, indicating gut ecosystem remodeling in response to chronic dietary stress and aging. Another notable observation indicates that some changes in the ApoA-IV-KO microbiome with the HFD also occur in WT mice, but with a delay, such as a decreased representation of *Akkermansia* and unclassified *Verrucomicrobiae* or increased abundance of unclassified *Bacilli.* At a late age (week 84), we observed a convergence of microbiota composition between ApoA-IV-KO and WT mice on the ND, supporting age-related microbiota homogenization [77]. Also, only a few bacteria differentiate ApoA-IV-KO and WT mice on the HFD. Nevertheless, significant differences in metabolite profiles persisted at week 84 under the HFD, including reduced BCAA and alanine levels in ApoA-IV-KO mice. Age-related changes in host metabolism are often mirrored by shifts in microbial metabolite production [78]. Elevated circulating levels of BCAAs have been linked to insulin resistance and metabolic aging and are frequently observed in obese and insulin-resistant individuals [2]. Interestingly, our observation of reduced BCAA levels in aged ApoA-IV-KO mice may reflect altered microbial fermentation patterns or host–microbiota interactions under chronic dietary stress. This is consistent with findings indicating that the microbial regulation of amino acid metabolism plays a key role in shaping host metabolic pathways during aging [79].

Similarly to microbiota composition, genotype-based comparisons (ApoA-IV-KO vs. WT) showed fewer differences in metabolite profiles than diet-based comparisons (HFD vs. ND), indicating that dietary factors exert a greater influence on metabolism than ApoA-IV deficiency. The most pronounced HFD-related differences involved both SCFAs, including a decrease in propanoic, isobutyric and butanoic acid levels, and several amino acids, especially an increase in BCAA levels. These findings align with earlier reports associating HFDs with altered amino acid metabolism and an increased risk of insulin resistance [80,81]. In WT mice, these changes were observed starting at week 28 and persisted to week 84, whereas in ApoA-IV-KO mice, this dominantly occurred at week 12. In contrast, genotype-related differences were limited and emerged mainly in old age. ApoA-IV-KO mice on the HFD displayed strong positive associations between SCFAs and the bacterial families *Pasteurellaceae*, *Staphylococcaceae*, and *Enterococcaceae*, suggesting compensatory microbiota mechanisms in the absence of ApoA-IV.

The interpretation of these results should consider several limitations inherent to this study. First, although the ApoA-IV-KO mouse model provides valuable insights into the metabolic and microbial consequences of ApoA-IV deficiency, the findings may not be directly translatable to humans due to interspecies differences in physiology and microbiota composition. Second, our analysis was limited to a single mouse strain (C57BL/6J), which may influence the observed phenotypes. Previous studies have shown that genetic background can significantly affect metabolic outcomes in ApoA-IV-deficient mice [62]. Furthermore, the associated lack of ApoC-III in the ApoA-IV-KO model should also be considered [2]. Next, while we observed clear associations between genotype, age, diet, microbiota composition, and metabolite profiles, this study was not designed to establish causality. Further mechanistic experiments, such as microbiota transplantation or targeted metabolite interventions, would be needed to confirm the functional relevance of these associations. Finally, only male mice were included in this study. Given known sex-related differences in metabolism and microbiota, and sex-dependent responses to HFDs in mice [70], future studies should include both sexes to fully capture biological variability. Despite these limitations, the longitudinal design and integration of microbiota and metabolomic data provide a robust framework for understanding the complex interactions between host genotype, diet, aging, and gut microbial ecology.

## 5. Conclusions

Our findings provided novel insights into age-, diet-, and genotype-related changes in microbiota composition and metabolite profiles, contributing to the growing body of evidence highlighting the central role of the gut microbiota in regulating metabolic, immune, and aging processes [82]. Diet exerted a stronger influence on both microbiota composition and metabolite profiles than genotype, with high-fat feeding inducing pronounced and persistent alterations across the lifespan. ApoA-IV deficiency modulated the timing of these changes and shaped distinct responses to dietary stress. Despite age-related convergence in microbiota structure, genotype-specific differences in metabolite profiles and SCFA–bacteria correlations persisted into old age, indicating a lasting impact of ApoA-IV on host metabolic adaptation. These findings underscore the importance of personalized dietary strategies and genetic screening in mitigating age-related metabolic disorders. Future research should explore the translational potential of modulating the gut microbiota and metabolite profiles through targeted interventions, such as prebiotics, probiotics, or dietary modifications, particularly in genetically predisposed populations. Moreover, integrating microbiome-based biomarkers into public health frameworks may enhance early detection and prevention strategies for metabolic and inflammatory diseases, ultimately contributing to healthier aging trajectories.

## Data Availability

Deep sequencing data were deposited at a European Bioinformatics Institute Metagenomics repository under accession number PRJNA1298785, according to 16S Metagenomic Sequencing.

## Supplementary Materials

The following supporting information can be downloaded at https://www.mdpi.com/article/10.3390/biology14091278/s1, Figure S1: Shannon and Chao indexes in comparison between HFD vs. ND groups; Figure S2: Shannon and Chao indexes in comparison between ApoA-IV vs. WT mice; Figure S3: Firmicutes to Bacteroidota ratio; Table S1: Microbiome comparison of ApoA-IV-KO vs. WT mice on ND (*p*_adj_ ≤ 0.05); Table S2: Microbiome comparison of ApoA-IV-KO vs. WT mice on HFD (*p*_adj_ ≤ 0.05); Table S3: Microbiome comparison of HFD vs. ND for ApoA-IV-KO mice (*p*_adj_ ≤ 0.05); Table S4: Microbiome comparison of HFD vs. ND for WT mice (*p*_adj_ ≤ 0.05).

## Abbreviations

The following abbreviations are used in this manuscript:

ApoA-IV	Apolipoprotein A-IV
HFDJ	High-fat diet
CCK	Cholecystokinin
KO	Knockout
ND	Normal diet
SCFA	Short-chain fatty acid
BCAA	Branched-chain amino acid
rCCA	Regularized canonical correlation analysis
WT	Wild type
GC/MS	Gas chromatography–mass spectrometry
F/B	Firmicutes to Bacteroidota
PCoA	Principal coordinate analysis
p_adj_	Adjusted *p*-value
