# Supplementary figures and images for: Lifetime Changes in Gut Microbiota and Metabolite Composition in High-Fat Diet-Induced Obesity in Apolipoprotein A-IV Gene Knockout Mice
Source: Biology (Basel). 2025 Sep 17;14(9):1278. doi: 10.3390/biology14091278 (PMC12467894; doi:10.3390/biology14091278)

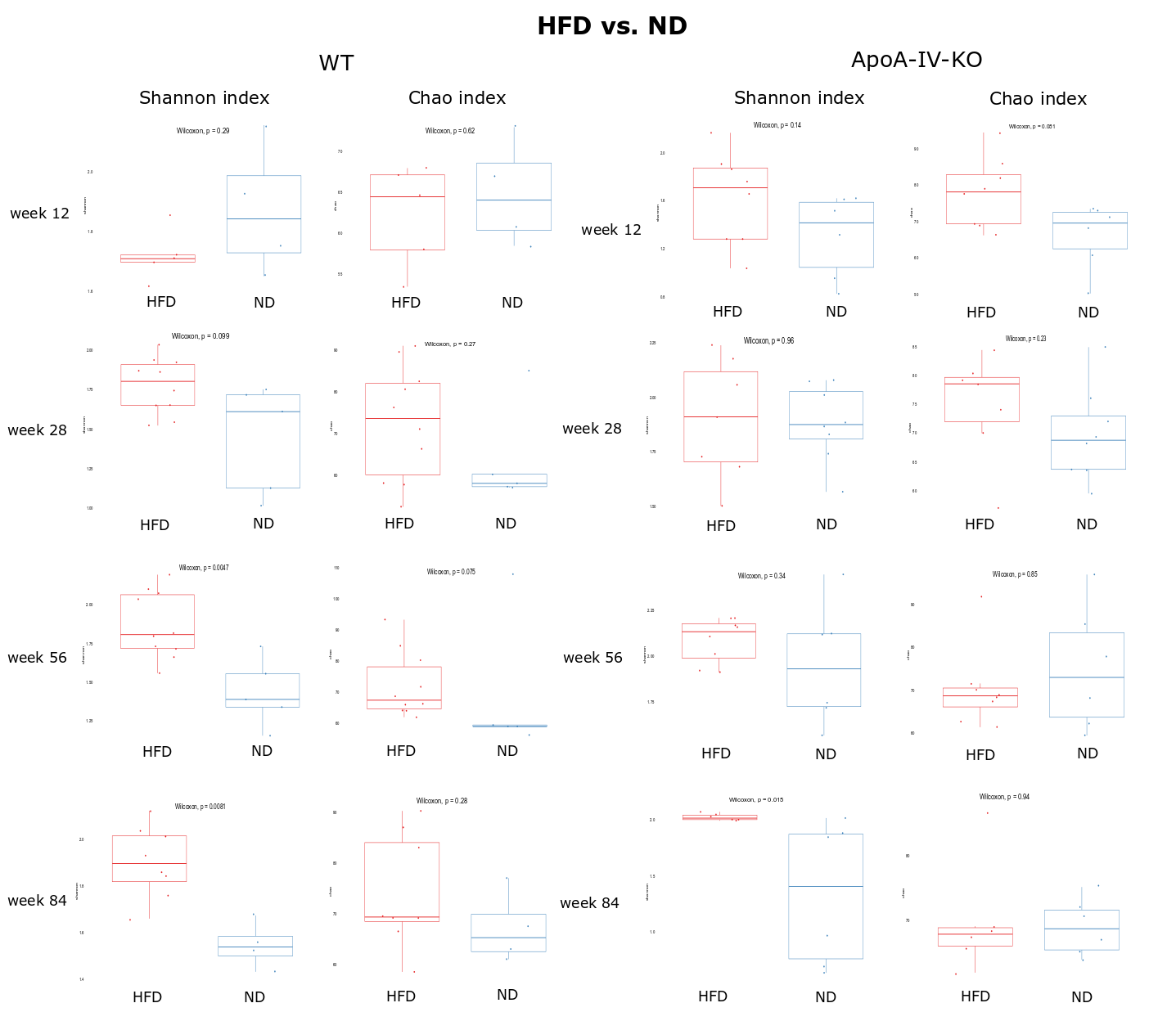

Supplement: Supplementary file 1 [file biology-14-01278-s001.zip › Figure S1 combo sh_ch_hfd vs nd.png]

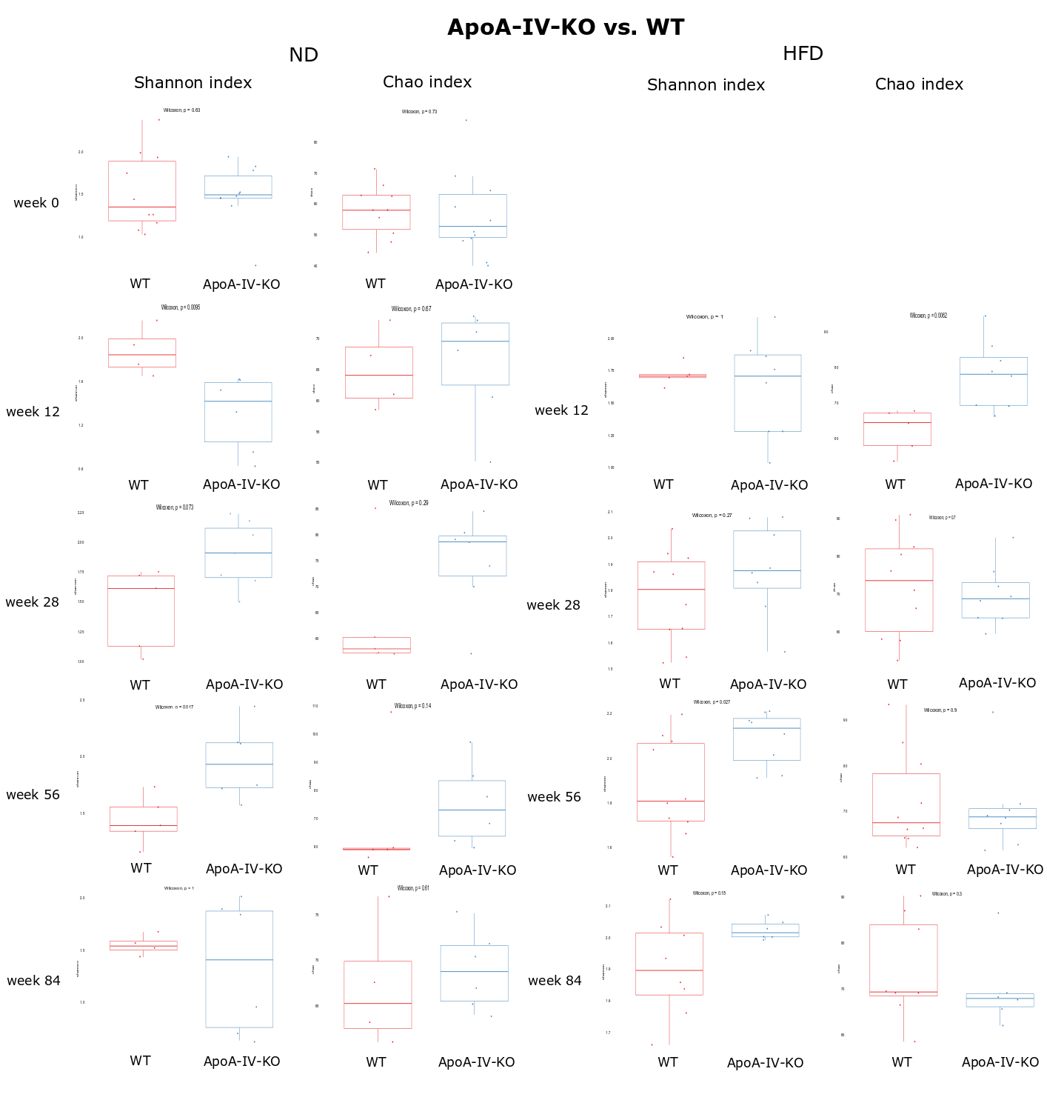

Supplement: Supplementary file 1 [file biology-14-01278-s001.zip › Figure S2 combo sh_ch_apo vs wt.png]

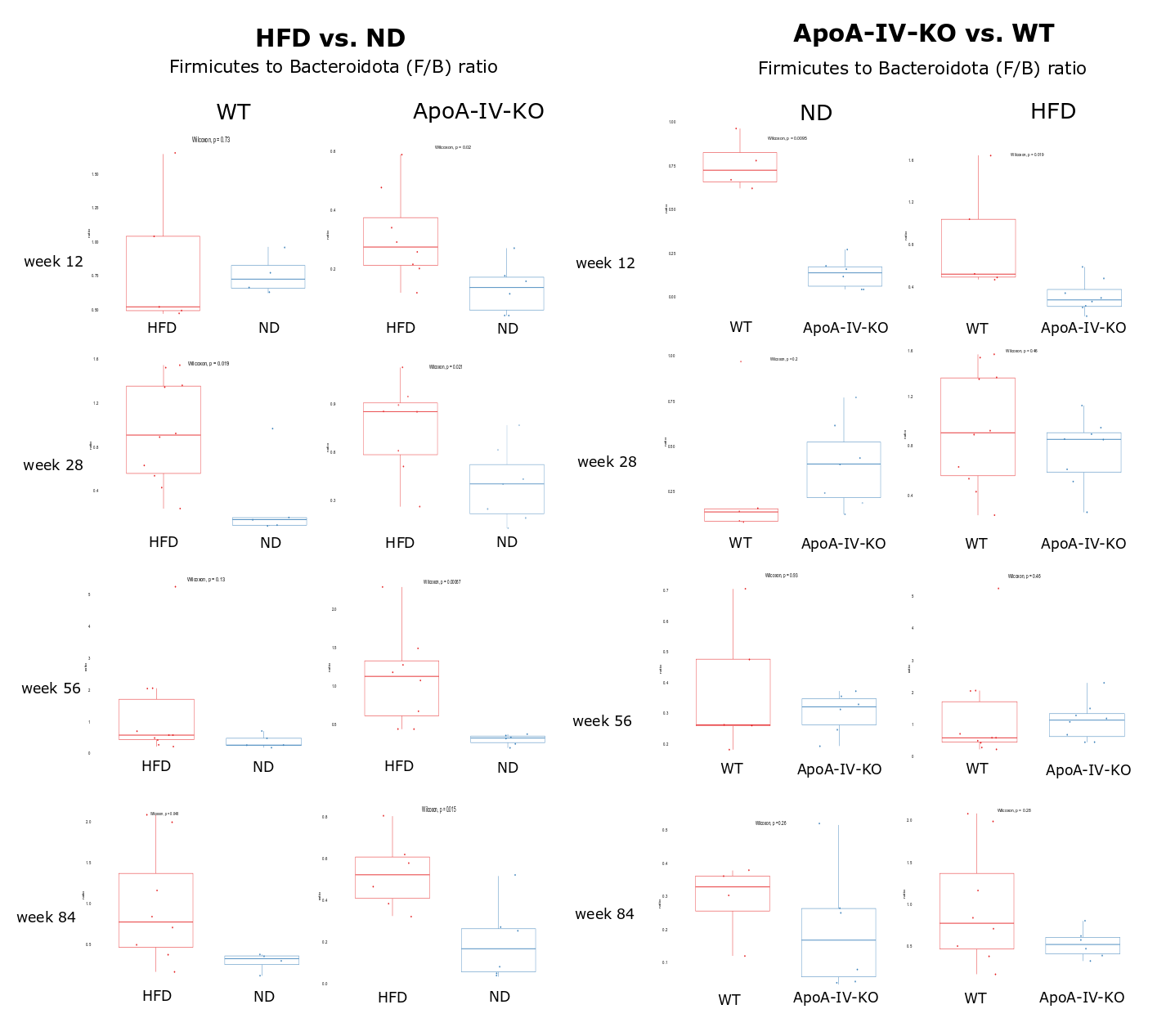

Supplement: Supplementary file 1 [file biology-14-01278-s001.zip › Figure S3 FB ratio combo.png]
